# Butyrate ingestion improves hepatic glycogen storage in the re-fed rat

**DOI:** 10.1186/1472-6793-8-19

**Published:** 2008-10-10

**Authors:** Marie-Christine Beauvieux, Hélène Roumes, Nadège Robert, Henri Gin, Vincent Rigalleau, Jean-Louis Gallis

**Affiliations:** 1 Centre de Résonance Magnétique des Systèmes Biologiques, UMR 5536 CNRS-UB2, 146 rue Léo Saignat, F-33076 Bordeaux Cedex France; 2 Service de Nutrition et Diabétologie, Hôpital Haut-Lévêque, Avenue de Magellan, F-33604 Pessac Cedex France

## Abstract

**Background:**

Butyrate naturally produced by intestinal fiber fermentation is the main nutrient for colonocytes, but the metabolic effect of the fraction reaching the liver is not totally known. After glycogen hepatic depletion in the 48-hour fasting rat, we monitored the effect of (butyrate 1.90 mg + glucose 14.0 mg)/g body weight *versus *isocaloric (glucose 18.2 mg/g) or isoglucidic (glucose 14.0 mg/g) control force-feeding on *in vivo *changes in hepatic glycogen and ATP contents evaluated *ex vivo *by NMR in the isolated and perfused liver.

**Results:**

The change in glycogen was biphasic with (i) an initial linear period where presence of butyrate in the diet increased (P = 0.05) the net synthesis rate (0.20 ± 0.01 μmol/min.g^-1 ^liver wet weight, n = 15) *versus *glucose 14.0 mg/g only (0.16 ± 0.01 μmol/min.g^-1 ^liver ww, n = 14), and (ii) a plateau of glycogen store followed by a depletion. Butyrate delayed the establishment of the equilibrium between glycogenosynthetic and glycogenolytic fluxes from the 6^th ^to 8^th ^hour post-feeding. The maximal glycogen content was then 97.27 ± 10.59 μmol/g liver ww (n = 7) at the 8^th ^hour, which was significantly higher than with the isocaloric control diet (64.34 ± 8.49 μmol/g, n = 12, P = 0.03) and the isoglucidic control one (49.11 ± 6.35 μmol/g liver ww, n = 6, P = 0.003). After butyrate ingestion, ATP content increased from 0.95 ± 0.29 to a plateau of 2.14 ± 0.23 μmol/g liver ww at the 8^th ^hour post-feeding (n = 8) [P = 0.04 *versus *isoglucidic control diet (1.45 ± 0.19 μmol/g, n = 8) but was not different from the isocaloric control diet (1.70 ± 0.18 μmol/g, n = 12)].

**Conclusion:**

The main hepatic effect of butyrate is a sparing effect on glycogen storage explained (i) by competition between butyrate and glucose oxidation, glucose being preferentially directed to glycogenosynthesis during the post-prandial state; and (ii) by a likely reduced glycogenolysis from the newly synthesized glycogen. This first demonstration of the improvement of liver glycogen storage by acute butyrate supply may be an important contribution to explaining the beneficial effects on glucose homeostasis of nutritional supply increasing butyrate amount such as fiber diets.

## Background

Carbohydrates are stored as glycogen in both muscles and the liver. As the first relay station for processing dietary information, the liver contains the whole biochemical machinery for both glucose and lipid storage and disposal [1]. Liver glycogen is the first defensive line when glycemia falls, and is thus the major source of circulating glucose except within 2–4 hours after a meal, the so-called postprandial period. These metabolic ways are controlled by the insulin level, insulin having several hepatic effects such as stimulation of glycogenosynthesis and glycolysis. The net effect is clear: when the supply of glucose is abundant, insulin "tells" the liver to bank as much of it as possible for subsequent use. In the absence of insulin, liver glycogenosynthesis ceases and the enzymes responsible for glycogen breakdown become active.

It has been proposed that the hepatic glycogen store may be influenced by lipids [2]. Reduced hepatic glycogen synthesis is thus an important characteristic of type 1 [3], type 2 [4] and MODY – 2 [5] diabetes in humans. Although long-chain free fatty acid are well known to induce peripheral insulin resistance and reduce muscle glycogenosynthesis [6], they have been reported to favor the sparing of hepatic glycogen in fasting humans [7], this increase being counterbalanced by acipimox [8], which inhibits lipolysis in peripheral tissues and induces a large reduction in circulating serum-free fatty acids. On the other hand, it has recently been reported that isolated liver perfusion with saturated or unsaturated FFAs reduced insulin signaling protein phosphorylation without affecting glycogen content [9]. It has been suggested that differential action of FFAs on glucose homeostasis could be linked to their nature [10]. In fact, peroxisome proliferator-activated receptor-α(PPAR-α), which is highly expressed in the liver, regulates fat metabolism and improves insulin sensitivity. Polyunsaturated FFAs are stronger inducers of PPAR-α activation than saturated FFAs [11]. It is unknown whether some effects may occur with short-chain fatty acids (SCFA) such as butyrate. Butyrate is a natural nutriment physiologically produced from intestinal fiber fermentation and found in foods such as butter. Butyrate is also the main nutriment for colonocytes. SCFA are used in artificial nutrition to allow successful transition to enteral feeding by maximizing the intestinal absorptive area in short bowel syndrome [12]. Moreover, recent studies on previously undescribed butyrate-producing bacteria from the human colon will help to unravel the effects of diet upon health, including microbial interactions with the immune system, and will help in the design of prebiotic or probiotic strategies for stimulating sub-optimal butyrate synthesis in the large intestine [13]. Besides the local effect, the remaining fraction reaches the liver through the portal vein to be metabolized. An almost 100% removal of butyrate by the liver has thus been evidenced in rats adapted to a high-fiber diet [14], suggesting better knowledge of the butyrate hepatic metabolism.

We recently reported a positive linear insulin- and glucose-dependent relationship between the net fluxes of glycogen and ATP in isolated perfused livers of fed rats [15]. This led us to hypothesize that some specific nutrients could interfere with hepatic glycogen synthesis, in that they modify the liver ATP content. Butyrate induced some changes in energetic metabolism such as (i) a rapid increase in the net rate of liver ATP consumption obtained *ex vivo *in the isolated and perfused liver [16], and (ii) a 77% decrease in the oxidative phosphorylation yield whereas respiration was greatly stimulated [17]. However, it is unknown whether butyrate influences the hepatic glycogen content. The purpose of the present study was therefore to explore the effect of butyrate used as a nutrient on *in vivo *ATP metabolism and glycogen storage in rat liver. After glycogen hepatic depletion by prolonged fasting, we used NMR to investigate the effect of force-feeding with glucose alone or glucose *plus *butyrate, in isocaloric and isoglucidic conditions, on the kinetic of (i) liver glycogen repletion and (ii) the change in ATP content. The *ex vivo *measurements were performed on the perfused and isolated organ, excised at different times post force-feeding.

## Results

### Kinetic of liver glycogen repletion after feeding with different diets

#### 1/Validation of NMR measurement

**Figure 1:**
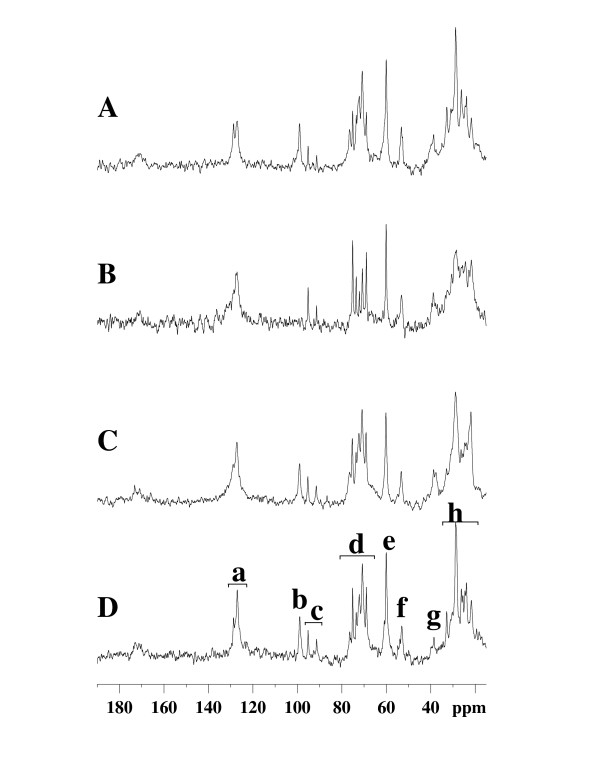
**Natural abundance ^13^C NMR typical spectra of isolated livers from rats in different nutritional conditions**. Livers were perfused and isolated from a rat (A) fed *ad libitum*, (B) starved for 48 hr, (C) 6^th ^hour post force-feeding with glucose 18.2 mg/g body weight, following 48 hr of fasting and (D) 6^th ^hour post force-feeding with (glucose 14.0 mg + butyrate 1.90 mg)/g body weight, following 48 hr of fasting. An external silicone reference gives a resonance at 0 ppm. Peak assignments: (a and h) fatty acids chains; (b) C-1 glycogen; (c) C-1α and C-1β glucose (mainly exogenous glucose of the perfusate); (d) glucose and glycogen (C-3β, C-5β glucose, glycogen; C-2 glucose; C-3α glucose; C-2, C-5α glucose, C-5 glycogen; C-4αβ glucose, glycogen); (e) C-6 glucose, glycogen; (f) choline; (g) ethanolamine. The chemical shift scale δ is given in parts per million (ppm) according to: chemical shift (Hz) = δ (ppm) × A(MHz), A being the frequency of the spectrometer. The unit ppm is used owing to the order value (10^-6^) of a constant characterizing the chemical nature of the nucleus. This scale allows an easy comparison between spectra obtained in spectrometers operating at different magnetic fields.

**Figure 2:**
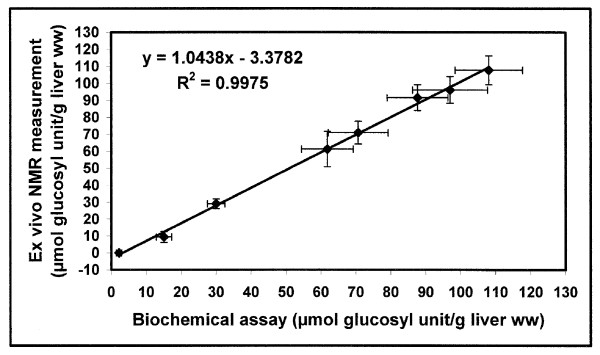
**Correlation between the liver glycogen content measured by NMR method and by biochemical assay**. m ± SEM. n varied from 4 to 6 for each mean. *Ex vivo *NMR measurement was performed on isolated perfused livers. Biochemical assay was performed on their corresponding perchloric extracts.

Liver glycogen content of rats fed *ad libitum *was 75 ± 8 μmol/g liver wet weight (ww) under glucose+insulin perfusion (n = 12) (Fig 1A). The ^13^C C-1 glycogen signal was not detected in the liver after 48 hr of starvation (Fig 1B) since ^13^C NMR sensitivity was limited to about 1 μmol/liver.g ww. To validate NMR measurements, we compared them with enzymatic analyses on the liver extracts (Fig 2). In fasting animals, the liver glycogen content determined by biochemical assay was 0.85 ± 0.34 μmol/g liver ww (n = 5). There was also a decrease in hepatic fatty acid content after 48 hr starvation, as previously reported [18].

Whatever the diet used, glycogen content increased regularly with time after the first 30 min post force-feeding. Glycogen content and time evolution were not different between *in vitro *enzymatic measurements and *ex vivo *NMR experiments. Two typical ^13^C NMR spectra of liver isolated at the 6^th ^hour post feeding with glucose alone or glucose+butyrate are shown in Fig. 1C and 1D, respectively. An increase in fatty acid content was also observed after feeding, as already described [18].

#### 2/diet A: 14.0 mg glucose/g body weight (Fig. 3)

**Figure 3:**
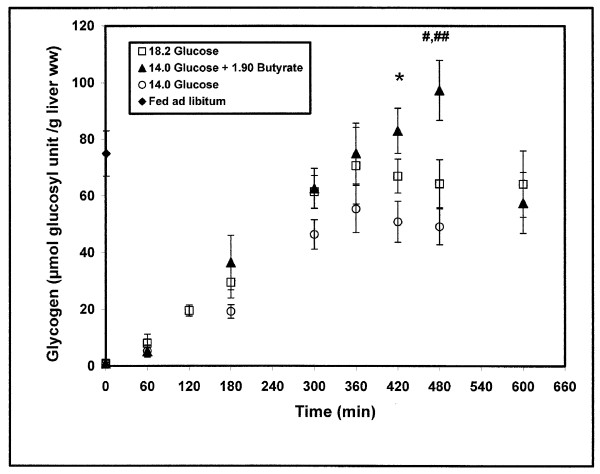
**Effect of the presence of butyrate in the diet on the kinetic of *in vivo *liver glycogen repletion after isoglucidic or isocaloric force-feedings of re-fed rats**. m ± SEM. n varied from 6 to 12 for each delay. *P = 0.01 (t-test) at the 7^th ^hour, diet A (glucose 14.0) *versus *diet C (glucose 14.0 + butyrate 1.90), # P = 0.003 at the 8^th ^hour, diet A (glucose 14.0) *versus *diet C (glucose 14.0 + butyrate 1.90) and ## P = 0.03 at the 8^th ^hour, diet B (glucose 18.2) *versus *diet C (glucose 14.0 + butyrate 1.90). Diets are expressed in mg/g body weight.

Starting with an undetectable NMR glycogen content in the fasting state, a time-dependent linear increase (0.16 ± 0.01 μmol/min.g^-1 ^liver ww) was observed during 300 minutes post feeding. Then a steady state of 55.39 ± 8.38 μmol/g liver ww (n = 6) was reached between the 5^th ^and the 6^th ^hour. Thereafter, the glycogen content slowly decreased (-0.052 ± 0.005 μmol/min. g^-1 ^liver ww) to reach 49.11 ± 6.35 μmol/g liver ww (n = 6) at the 8^th ^hour post feeding.

#### 3/diet B: 18.2 mg glucose/g body weight (Fig. 3)

From the NMR undetectable glycogen content in the fasting state, a time-dependent linear increase (0.20 ± 0.01 μmol/min.g^-1 ^liver ww) was observed during 300 minutes post feeding. A maximal level of 70.63 ± 13.52 μmol/g liver ww (n = 10) was reached at the 6^th ^hour. Thereafter, the glycogen level slowly decreased (-0.025 ± 0.002 μmol/min.g^-1 ^liver ww) nearly 2-fold more than in diet A, to reach 64.24 ± 11.68 μmol/g liver ww (n = 9) at the 10^th ^hour post feeding.

#### 4/diet C: (14.0 mg glucose + 1.90 mg butyrate)/g body weight (Fig. 3)

Diets A and C were isoglucidic, while diets B and C were isocaloric (7.28 cal), diet C containing less glucose (-23%) than diet B. After ingestion of diet C containing butyrate, a linear increase in glycogen content (0.20 ± 0.01 μmol/min.g^-1 ^liver ww, higher than in isoglucidic diet, P = 0.05) was observed over 8 hours at the same rate as in isocaloric diet B, despite the slightest glucose content. A maximal glycogen level of 97.27 ± 10.59 μmol/g liver ww (n = 7) was reached at the 8^th ^hour post feeding, which was significantly higher (P = 0.03) than in isocaloric diet B (64.34 ± 8.49 μmol/g liver ww, n = 12) or isoglucidic diet A (P = 0.003). Thereafter, the glycogen content rapidly decreased at a rate of -0.33 ± 0.04 μmol/min.g^-1 ^ww to reach a level of 57.59 ± 17.80 μmol/g liver ww at the 10^th ^hour (n = 9), a value similar to that in isocaloric diet B.

#### 5/Areas Under the Curves

**Figure 4:**
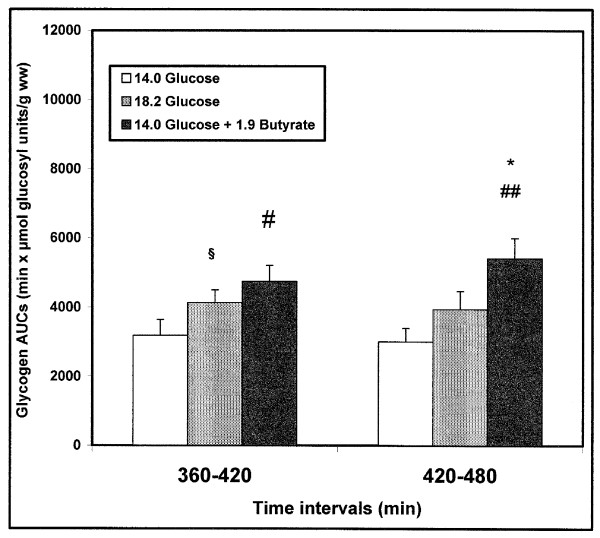
**Areas Under the Curves of *in vivo *liver glycogen repletion for time intervals of interest in re-fed rats**. m ± SEM. n varied from 6 to 10 for each delay. Diet B (18.2 mg glucose/g) *versus *diet A (14.0 mg glucose/g): §P = 0.05. Diet C (14.0 mg glucose/g + 1.90 mg butyrate/g) *versus *diet A (14.0 mg glucose/g): # P = 0.02, ## P = 0.004. Diet C (14.0 mg glucose/g + 1.90 mg butyrate/g) *versus *diet B (18.2 mg glucose/g): * P = 0.04. Diets are expressed in mg/g body weight.

Areas under the curve (AUCs) highlighted the effects of glucose and butyrate contents in diet on the glycogen resynthesis. A significant increase (P = 0.04) was obtained in AUC Glycogen_0–420 _in diet B (18.2 mg glucose/g) *versus *diet A (14.0 mg glucose/g). A significant increase in AUCs glycogen in diet C (14.0 mg glucose/g + 1.90 mg butyrate/g) *versus *diet A (14 mg glucose/g) was obtained: AUC Glycogen_0–360 _P = 0.05, AUC Glycogen_0–420 _P = 0.02, AUC Glycogen_0–480 _P = 0.015. Significant higher values of AUCs glycogen in diet C *versus *diets A and/or B at particular periods of interest (360–420 min and 420–480 min) are presented (Fig 4).

### Kinetic of liver ATP content after feeding with the different mixtures (Fig. 5)

**Figure 5:**
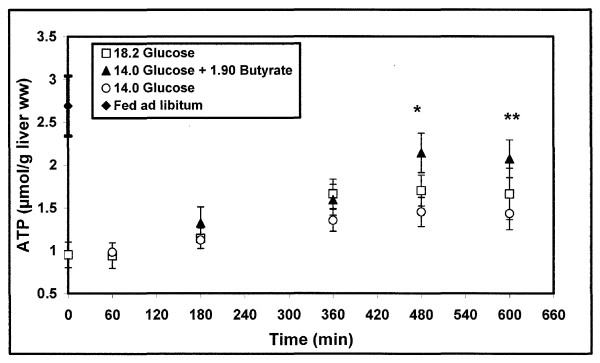
**Kinetic of *in vivo *liver ATP resynthesis after different force-feedings of re-fed rats**. m ± SEM. n varied from 6 to 12 for each delay. *P = 0.04 at the 8^th ^hour and **P = 0.05 at the 10^th ^hour (t-test), diet C (glucose 14.0 mg + butyrate 1.90 mg) *versus *diet A (glucose 14.0 mg). Diets are expressed in mg/g body weight.

The ATP level was 2.69 ± 0.35 μmol/g liver ww (n = 12) in rats fed *ad libitum *and 0.95 ± 0.29 μmol/g liver ww after 48 hr fasting (n = 5). Whatever the diet, the ATP content remained unchanged during the first hour after feeding.

#### 1/diet A: 14.0 mg glucose/g body weight

The ATP content slightly increased (1.2 ± 0.2 10^-3 ^μmol/min.g^-1 ^liver ww) after the 1^st ^hour post feeding, from the basal state to a plateau of 1.35 ± 0.17 μmol/g liver ww (n = 6) reached at the 6^th ^hour.

#### 2/diet B: 18.2 mg glucose/g body weight

The ATP time change was similar to that in diet A, except that the ATP net flux was 2.3 ± 0.4 10^-3 ^μmol/min.g^-1 ^liver ww; ATP level reached a plateau of 1.66 ± 0.29 μmol/g liver ww (n = 10) at the 6^th ^hour. Thereafter, it remained stable until the 10^th ^hour post force-feeding.

#### 3/diet C: (14.0 mg glucose + 1.90 mg butyrate)/g body weight

From the 1^st ^hour post force-feeding, the ATP content slightly increased (2.5 ± 0.2.10^-3 ^μmol/min.g^-1 ^liver ww) to reach a plateau of 2.14 ± 0.23 μmol/g liver ww at the 8^th ^hour post feeding (n = 8) [P = 0.04 *versus *isoglucidic diet A (1.45 ± 0.19 μmol/g liver ww, n = 8), but this was not different from isocaloric diet B (1.70 ± 0.18 μmol/g liver ww, n = 12)]. The ATP content remained stable until the 10^th ^hour post feeding, the presence of butyrate in the diet inducing a higher ATP level than after the isoglucidic diet A (P = 0.05).

### Changes in serum glucose and insulin concentration at the steady state

In the 48 hr-fasted rats, glycemia and insulinemia were 4.9 ± 0.4 mmol/L and 13.8 ± 1.9 μUI/L, respectively (n = 5). In the fed rats, glycemia was similar with the glucose+butyrate diet C (10.25 ± 0.8 mmol/L, n = 4) and isocaloric glucose diet B (9.5 ± 0.5 mmol/L, n = 4) at the plateau of glycogen content, whereas insulinemia was lower (P = 0.04) with the glucose+butyrate diet (17.3 ± 2.3 μUI/L, n = 4) than with the isocaloric glucose diet (23.1 ± 3.1 μUI/L, n = 4). Although we did not directly measure insulin sensitivity, this decrease in insulin concentration whereas glycemia was maintained suggested an improved insulin sensitivity. Hence, according to the glycemia-to-insulinemia ratio, the peripheral insulin sensitivity was significantly higher at the steady state of liver glycogen content after feeding with butyrate (diet C) (6.44 ± 0.79 AU, n = 4) compared to the isocaloric glucose diet B (4.32 ± 0.56 AU, n = 4, P = 0.009) and to the fasting state (3.55 ± 0.43 AU, n = 5, P = 0.011).

## Discussion

The initial purpose of this study was to explore the effect of butyrate, used as a nutrient, on *in vivo *glycogen storage and ATP content in the rat liver. The *ex vivo *evaluations of the contents were performed on the perfused and isolated organ excised at different times after force-feedings, to accurately reflect the *in vivo *metabolic contents at each time. In order to mimic physiological conditions and to avoid a rapid and dramatic decrease of the energetic metabolism linked to ischemia, the liver was perfused as previously described [17,19]. The presence of glucose 30 mM and insulin reproduced the post-prandial state in the portal vein and allowed to maintain the carbohydrate metabolism [15]. We sought to investigate the effect of short-chain FA on hepatic glycogen, since contradictory effects on glycogen storage have been previously reported for long chain FA [8,9]. Even if propionate, another end-product of colonic bacterial fermentation, could be an interesting candidate to study the metabolism of glycogen, the role of this SCFA as a gluconeogenic precursor would be confusing in this study focused on butyrate (i) since it is not directly a gluconeogenic substrate and (ii) is known to directly decrease the *ex vivo *ATP production [16,17] affecting thus perhaps the glycogen pathway.

To our knowledge, few studies focused on the kinetic of *in vivo *liver glycogen resynthesis in fasting rats over a wide time range (10 hrs post feeding). Since total glycogen depletion is an essential factor to evaluate the initial rate of glycogen resynthesis, we utilized 48-hr starvation in order to empty the glycogen store because 24-hr starvation does not totally deplete it [20]. Although 48-hr starvation has been reported to increase serum FFA [21] which may interfere with the action of insulin, the duration of fasting in the present work did not differ between all experimental groups. The animals were force-fed once to be sure (i) that all the substrates were ingested and (ii) that the acute effect of butyrate could be investigated since absorption of the colonic butyrate was spread over 24 hr, whilst force-fed butyrate was absorbed within a few hours. Moreover, this intake way avoided the repulsion linked to its taste. It is known that this model allows to rigorously control the animal's consumption of dietary nutrients [22].

The quantities of butyrate administered (1.90 mg/g body weight equal to 190 mg for a 100 g body weighted rat) are not very different from those likely to be produced with a 10% fiber diet or reported to be produced by bacterial fermentation of dietary fiber in the rats [23]. However, owing to the acidic pH conditions in the stomach accelerating butyrate absorption as protonated form, and if gastric epithelium permeability was elevated, the fraction reaching the liver could be higher than those arising from colonic fermentation. In fact, as shown in preliminary report [24], a change of ATP turnover (with maintain of ATP content) in the same experimental conditions was observed 2–3 hr only after the butyrate force-feeding, suggesting that its gastric absorption was not dramatically rapid.

We distinguished two main phases in the glycogen-storage process that varied with the presence or absence of butyrate in the diet: (i) an initial period of glycogen repletion and (ii) a plateau of glycogen content followed by depletion.

Glycogenosynthesis and glycolysis are the two main modes of glucose disposal in the liver. Glycogen repletion showed the well-known glucose dose-dependent increase in glycogen synthesis, as evidenced with the two different doses of glucose alone as already reported [20].

Surprisingly, a similar hepatic glycogen synthesis rate (about 20 μmol/min/g ww) was obtained with (i) the diet containing 18.2 mg glucose/g body weight and (ii) the isocaloric diet containing butyrate and a lower concentration of glucose (14.0 mg/g). To our knowledge there is no experimental evidence to date a direct stimulation of glycogenosynthesis pathway by butyrate. However in fed rat, the stimulation of liver lactate utilization by various fatty acids has been ascribed to a stimulation of gluconeogenesis [25,26] and thus the contribution of this latter to glycogenosynthesis cannot be excluded. The other mode of glucose disposal is its oxidation, so 14.0 mg glucose/g could theoretically lead to fewer fluxes of both glycolysis and mitochondrial ATP production than the oxidation of 18.2 mg glucose/g. The observation of a similar hepatic net flux of ATP production with 18.2 mg glucose/g and (14.0 mg glucose/g + 1.90 mg butyrate/g) suggests that butyrate oxidation (within β-oxidation and the tri-carboxylic cycle) partly replaced glucose as fuel in the oxidation processes, leading to ATP through mitochondrial oxidative phosphorylation. Some of the ATP could be used for glycogenosynthesis. Moreover, ATP production from glucose oxidation was probably reduced since fatty acids are generally known to inhibit glycolysis [27], as reported for butyrate in isolated hepatocytes [28]. Hence it may be concluded that butyrate induced a hepatic glycogen-sparing effect by reducing glycolysis, the glucose being exclusively involved in glycogenosynthesis during the first 6 hours post feeding. This result is in agreement with a previous report concerning parenteral nutrition in rats in which the combinations of glucose and fat had a sparing effect on body fat and hepatic glycogen [29].

Another mode of glycogen saving could be that butyrate spares the glycogen store *via *inhibition of the glycogenolytic flux through two mechanisms: (i) a putative direct inhibitory effect of butyrate on glycogenolysis and (ii) a metabolic effect through ATP production, as ATP is known to have an inhibitory effect on liver phosphorylase A (and thus on glycogenolysis) [30]. However, the similar ATP levels and the similar net ATP synthesis rate observed during the first hours following feeding with or without butyrate (in isocaloric conditions) are not in favor of this second hypothesis. After the glycogen repletion step, liver glycogen content reached a steady state evidencing an equilibrium between the unidirectional glycogenosynthesis flux and the unidirectional glycogenolysis flux. Importantly, butyrate delayed the establishment of the flux equilibrium with a higher net glycogen content until the 8^th ^hr post force-feeding. This can only be due to (i) the maintenance of glycogenosynthesis and (ii) the inhibition of glycogenolysis, in agreement with recent data showing that in type 2 diabetes mellitus patients, free fatty acids improve glucoregulation merely through modulation of the rate of glycogenolysis [31]. Further studies are required to confirm the latter hypothesis. A better understanding of the mechanistic aspects of butyrate hepatic effect may implied evaluation of changes of uridine diphosphate (UDP)-glucose, glucose-1-phosphate (G1P) or glucose-6-phosphate (G6P), as important metabolic crossroads of the glycogen pathways. Indeed, hepatic glycogen post-prandial synthesis can be estimated through the level of UDP-glucose, which requires mass spectroscopy [32,33]. Moreover, metabolic conditions altering i.e. the hepatic G6P content were reported to affect glycogen metabolism through enzymatic regulation [34]. However, natural abundance NMR spectra in isolated liver were not sufficiently resolutive to accurately discriminate and quantify UDP-glucose and phosphorylated monosaccharides.

Butyrate is physiologically produced by the microflora from the fermentation of dietary fiber and mainly has a trophic effect. Owing to the capacity of the liver to remove incoming propionate and butyrate, the hepatic utilization of the latter is proportional to the digestive supply [14,35]. Moreover, since butyrate is almost totally removed in animals on a very high fiber diet (35%) [14], our data suggest that dietary fiber may have a metabolic effect on the glucose and glycogen hepatic pathways.

This effect should be added to the known intestinal mechanical effect of fiber that delays the gastrointestinal action of amylase. Human studies have already shown that the glycemic index of carbohydrates is not solely based on different degrees of digestive absorption, but may also depend on changes in glucose disposal [36]. Intake of dietary fibers has been reported to be associated (i) inversely with the probability of having protection against insulin resistance in non-diabetic patients [37] and (ii) with enhanced insulin sensitivity in type 2 diabetes, whatever the type of fiber [38]. Rather than a low total carbohydrate diet, a balanced carbohydrate intake rich in dietary fiber such as high fruit and vegetable intake [39] or whole grain cereal products [40] could be protective against metabolic syndrome. Accordingly, the present findings demonstrate a possible increase in insulin sensitivity in the interprandial period after feeding with butyrate. The low frequency of hypoglycemic events following a long-term high-fiber diet (50 g/day) [41] may also be linked to the higher hepatic glycogen content induced by the time cumulative effect of butyrate.

More anecdotaly, butyrate effect should in part contribute to the effect of acarbose used in the pharmacological prevention of diabetes. Indeed, in rats fed with "Westernized" diets, acarbose treatment suppressed starch digestion in the small bowel but there was compensatory salvage by bacterial fermentation in the large bowel. Cecal total SCFA pool size was increased more than 4-fold, with even bigger increases for butyrate. These changes in butyrate were reflected in increased molar proportions of butyrate in blood from both the portal vein and heart [42]. Acarbose could effectively increased colonic butyrate production by several mechanisms such as reduced starch absorption and larger concentrations of starch-fermenting and butyrate-producing bacteria [43].

## Conclusion

It must be kept in mind that under our experimental conditions (acute butyrate supply and gastric absorption), the observed effects could not be strictly transposed to fiber diets. Whatever the case, the hepatic sparing effect of butyrate on both glucose oxidation and glycogen store, could be one of the molecular basis for the effects of dietary fibers on the prevention of insulin resistance. In the muscle, the prevention of insulin resistance in rats supplemented in fiber has been explained by the effect of butyrate increasing GLUT-4 *via *PPARγ [44]. To our knowledge, our work is the first to propose a biochemical mechanism in the liver to better understand how one of the main end-products of dietary fibers may regulate glucose metabolism. Since these data were obtained after acute intake of butyrate, the chronic effects remain to be studied. The use of dietary fibers could be a simple, non-invasive and socially acceptable method to improve the metabolic pathways involved in metabolic syndrome [45].

## Methods

### Chemicals

High-grade chemicals were purchased from Sigma Chemical (St. Louis, Missouri, USA) except where otherwise specified.

### Animals

Male Wistar rats (Centre d'élevage Depré, St Doulchard, France) weighing 90–120 g were fed *ad libitum *with a balanced diet: carbohydrates (65%), proteins (16%), water (12%), minerals (5%), fibers (4%) and lipids (3%) amounting to 12.75 MJ/kg food. They were fasted for 48 hr, with free access to water, in order to deplete totally their hepatic glycogen store. A fasting period of 48 hr was currenly used in various protocols concerning liver metabolic studies in rats [46-48]. They were then force-fed with an intragastric bolus of one of the following mixtures: (i) diet A was 14.0 mg glucose/g body weight, (ii) diet B was 18.2 mg glucose/g body weight, or (iii) diet C was (14.0 mg glucose + 1.90 mg butyrate)/g body weight calculated to be isocaloric compared to diet B (7.28 cal whereas diet A was 5.60 cal) and isoglucidic compared to diet A. A significant difference (23%) was thus obtained in the glucidic energy supply. All mixtures were diluted with water for a total force-feeding volume of 1.8 ml/100 g body weight, respecting the maximal recommendations of 20 ml/kg of body weight. Intragastric administration with a cannula (Harvard apparatus; 16 gauge diameter; 4 inches long) was performed within 1 minute.

To determine the kinetic of *in vivo *hepatic ATP and glycogen contents, *ex vivo *NMR measurements were performed on isolated and perfused liver. For this purpose, animals were anesthetized at different times from the force-feeding time (t = 0) to 10 hours post force-feeding and the liver was perfused and then excised for immediate NMR measurements. n varied from 6 to 12 for each delay. Venous blood was collected, immediately centrifuged (20 minutes at 3500 g) and stored at -80°C in order to measure serum glucose and insulin. Immediately after the perfusion of the portal vein, the anesthethized rats were euthanasied by instant decapitation with scientific guillotine.

The laboratory is licensed for animal experiments (French Agriculture Department). The study complied with 1999 UFAW guidelines [Handbook on the Care and Management of Laboratory Animals Vol 1, 7th edn Terrestrial Vertebrates. Oxford (Poole T, English P)]. The protocol for these experiments was approved by the Regional Ethics Committee for Animal Experiment of Aquitaine-Poitou-Charentes.

### Liver perfusion

Media were diluted daily from concentrated stock solutions. Standard Krebs-Heinseleit (KHB) buffer was composed of (in mmol/L) 120 NaCl, 4.70 KCl, 1.20 MgSO_4_, 25 NaHCO_3_, 1.20 KH_2_PO_4_-K_2_HPO_4_, 1.30 CaCl_2_, 0.30 Na-pyruvate and 2.10 Na-lactate (pH = 7.35 at 37°C). Rats were anesthetized 1 hr, 2 hrs, 3 hrs, 4 hrs, 6 hrs, 8 hrs and 10 hrs after force-feeding by intraperitoneal injection of pentobarbital sodium (50 mg/kg of rat). The technique of liver antegrade perfusion through the portal vein, to mimic physiological conditions, was described previously [15-17,19]. Briefly, the liver (4–6 g) was perfused in normothermal and well-oxygenated conditions with KHB containing glucose (30 mmol/L) and insulin (120 mUI/L) (concentrations chosen to mimic the physiological post-prandial state in the portal trunk) [49,50] at 37°C regulated by a thermostatic bath. The perfusate was pumped through a Silastic^© ^home-made oxygenator, gassed with 95% O_2 _and 5% CO_2 _(1 bar pressure). Perfusion flow was kept constant (5 ml/min.g liver wet weight) and was sufficient to ensure good oxygenation of the liver. Perfusate temperature and pH were monitored both before entering and after leaving the liver by continuous-flow pH electrodes and temperature electrodes. The perfused liver was then excised from the rat abdomen and transferred to a 20 mm-diameter NMR cell for direct glycogen and ATP *ex vivo *measurement. Liver was then freeze-clamped for subsequent ethanol extraction for *in vitro *enzymatic determination of glycogen content and/or NMR determination.

### NMR methodology

The spectra were obtained using a ^31^P/^13^C double-tuned 20 mm probe operating at 9.4T. Liver ATP content was monitored by ^31^P NMR and carbohydrate content in natural abundance was assessed by ^13^C NMR. ^31^P and ^13^C NMR spectra were recorded at 161.9 and 100.6 MHz respectively on a DPX400 spectrometer (Brucker). The magnetic field was adjusted on the water proton signal. ^31^P NMR spectra were first obtained (100 free induction decays (FID); 1 min acquisition time) without proton decoupling. Radiofrequency pulses (70° flip angle) and 10,000 Hz spectral width were used for data acquisition. ^13^C NMR spectra were proton-decoupled using a gated bi-level mode. ^13^C NMR spectra were obtained (200 FID; 3 min acquisition time) from a 66° radiofrequency pulse repeated every second (25,000 Hz spectral width). Spectrometers with high magnetic field (9.4 T) allow the obtention of high resolutive and informative metabolites spectra. However, owing to the limited diameter of the probe (25 mm) of the spectrometer, the *in vivo *experiments on whole rat were excluded, hence accurate measurements were performed on the isolated and perfused liver.

Lorentzian line broadening of 15 Hz was applied before Fourier transformation for both ^31^P and ^13^C NMR spectra. Chemical shift of phosphorylated metabolites was expressed relative to the position of resonance in the frequency scale of an internal reference set (glycerophosphoryl-choline) at 0.47 ppm.^13^C chemical shifts were expressed from an external silicone reference (1.45 ppm) (the absolute quantity of hepatic glycogen concentration was calculated by comparing the peak integral with that of oyster glycogen standard [0 to 185 mM glycosyl units] obtained under identical conditions). During the first minutes of perfusion (total perfusion duration: 20 minutes), we discarded any liver showing an increase in the intensity of inorganic phosphate resonance occurring with a concomitant decrease in β NTP signals (corresponding to more than 80% of the total liver ATP), probably reflecting some partial lobe ischemia. ^13^C NMR analysis was performed only on liver showing a stable high β NTP level.

### Perchloric extraction

The freeze-clamped liver was quickly weighed and ground into liquid nitrogen. It was homogenized with 10 volumes of HCLO_4 _at 0°C and centrifuged (1000 g, 5 min, 4°C). The supernatant was neutralized with KOH in order to precipitate KCLO_4 _and was then centrifuged (1000 g, 5 min, 4°C). The supernatant was divided for (i) ^13^C NMR analysis of glycogen and (ii) for enzymatic analysis (glucose oxidase) performed after ethanol extraction. Finally, for each liver, the comparison between the glycogen content measured by (i) *ex vivo *NMR method on the perfused organ and (ii) the biochemical assay on its perchloric extract, evidenced a linear relationship (Fig 2).

### Glucose and insulin level determination

Blood (1 ml) was collected in the inferior vena cava just before liver perfusion. The blood was immediately centrifuged (20 min, 3500 g). Glycemia (expressed as mmol/L) was measured by hexokinase at 340 nm (multiparametric analyzer Olympus AU640, Tokyo, Japan) and insulinemia (expressed as μUI/L) by radioimmuno assay (Sanofi Diagnostic Pasteur, Marne la Coquette, France). The 10 × (glycemia/insulinemia) ratio was calculated to estimate insulin sensitivity [51]. The results were expressed in arbitrary units (AU) = 10 × (mmol/μUI).

### Results expression and Statistics

All results were expressed as means ± SEM. Different phases of glycogen resynthesis were represented by the AUCs. Statistical analysis was performed using one-way analysis of variance (ANOVA) for all data analysis. A t-test was performed following the ANOVA (P value lower than 0.05 was considered to be significant).

## Abbreviations

ATP: adenosine triphosphate; AU: arbitrary units; FFA: free fatty acids; KHB: Krebs Henseleit buffer; NMR: nuclear magnetic resonance; SCFA: short-chain free fatty acids; ppm: parts per million; ww: wet weight.

## Authors' contributions

MCB participated in the design and coordination of the study such as the experimental procedures and was highly involved in revising the manuscript. HR carried out many of the experimental procedures, performed data analysis and was involved in drafting the manuscript. NR carried out many of the experimental procedures and data analysis. HG was highly involved in drafting the manuscript. VR was involved in revising the manuscript. JLG designed the study, carried out the analysis and data interpretation and was involved in drafting the manuscript. All authors read and approved the final manuscript.

## References

[B1] Petruzzelli L, Lo Sasso G, Portincasa P, Palasciano G, Moschetta A (2007). Targeting the liver in the metabolic syndrome: evidence from animal models. Curr Pharm Des.

[B2] Stingl H, Krssak M, Krebs M, Bischof MG, Nowotny P, Furnsinn C, Shulman GI, Waldhaus W, Roden M (2001). Lipid-dependent control of hepatic glycogen stores in healthy humans. Diabetologia.

[B3] Hwang JH, Perseghin G, Rothman DL, Cline GW, Magnusson I, Petersen KF, Shulman GI (1995). Impaired net hepatic glycogen synthesis in insulin-dependent diabetic subjects during mixed meal ingestion. A ^13^C nuclear magnetic resonance spectroscopy study. J Clin Invest.

[B4] Krssak M, Brehm A, Bernroider E, Anderwald C, Nowotny P, Dalla Man C, Cobelli C, Cline GW, Shulman GI, Waldhausl W, Roden M (2004). Alterations in postprandial hepatic glycogen metabolism in type 2 diabetes. Diabetes.

[B5] Velho G, Petersen KF, Perseghin G, Hwang JH, Rothman DL, Pueyo ME, Cline GW, Froguel P, Shulman GI (1996). Impaired hepatic glycogen synthesis in glucokinase-deficient (MODY-2) subjects. J Clin Invest.

[B6] Petersen KF, Shulman GI (2006). Etiology of insulin resistance. Am J Med.

[B7] Muller C, Assimacopoulos-Jeannet F, Mosimann F, Schneiter P, Riou JP, Pachiaudi C, Felber JP, Jequier E, Jeanrenaud B, Tappy L (1997). Endogenous glucose production, gluconeogenesis and liver glycogen concentration in obese non-diabetic patients. Diabetologia.

[B8] Allick G, Sprangers F, Weverling GJ, Ackermans MT, Meijer AJ, Romijn JA, Endert E, Bisschop PH, Sauerwein HP (2004). Free fatty acids increase hepatic glycogen content in obese males. Metabolism.

[B9] Anderwald C, Brunmair B, Stadlbauer K, Krebs M, Fürnsinn C, Roden M (2007). Effects of free fatty acids on carbohydrate metabolism and insulin signalling in perfused rat liver. Eur J Clin Invest.

[B10] Han P, Zhang YY, Lu Y, He B, Zhang W, Xia F (2008). Effects of different free fatty acids on insulin resistance in rats. Hepatobiliary Pancreat Dis Int.

[B11] Clarke SD (2001). Nonalcoholic steatosis and steatohepatitis. I. Molecular mechanism for polyunsaturated fatty acid regulation of gene transcription. Am J Physiol Gastrointest Liver Physiol.

[B12] Bartholome AL, Albin DM, Baker DH, Holst JJ, Tappenden KA (2004). Supplementation of total parenteral nutrition with butyrate acutely increases structural aspects of intestinal adaptation after an 80% jejunoileal resection in neonatal piglets. J Parenter Enteral Nutr.

[B13] Pryde SE, Duncan SH, Hold GL, Stewart CS, Flint HJ (2002). The microbiology of butyrate formation in the human colon. FEMS Microbiol Lett.

[B14] Demigné C, Yacoub C, Rémésy C (1986). Effects of absorption of large amounts of volatile fatty acids on rat liver metabolism. J Nutr.

[B15] Baillet-Blanco L, Beauvieux MC, Gin H, Rigalleau V, Canioni P, Gallis JL (2005). Insulin induces a positive relationship between the rates of ATP and glycogen changes in isolated rat liver in presence of glucose; a 31P and 13C NMR study. Nutr Metab.

[B16] Beauvieux MC, Tissier P, Gin H, Canioni P, Gallis JL (2001). Butyrate impairs energy metabolism in isolated perfused liver of fed rats. J Nutr.

[B17] Gallis JL, Tissier P, Gin H, Beauvieux MC (2007). Decrease in oxidative phosphorylation yield in presence of butyrate in perfused liver isolated from fed rats. BMC Physiol.

[B18] Allmann DW, Hubbard DD, Gibson DM (1965). Fatty acids synthesis during fat-free refeeding of starved rats. J Lipid Res.

[B19] Delmas-Beauvieux MC, Gallis JL, Rousse N, Clerc M, Canioni P (1992). Phosphorus-31 nuclear magnetic resonance of isolated rat liver during hypothermic ischemia and subsequent normothermic perfusion. J Hepatol.

[B20] Shulman GI, Rothman DL, Smith D, Johnson CM, Blair JB, Shulman RG, DeFronzo RA (1985). Mechanism of liver glycogen repletion *in vivo *by nuclear magnetic resonance spectroscopy. J Clin Invest.

[B21] Kmiec Z, Pokrywka L, Kotlarz G, Kubasik J, Szutowicz A, Mylsliwski A (2005). Effects of fasting and refeeding on serum leptin, adiponectin and free fatty acid concentrations in young and old male rats. Gerontology.

[B22] Nanji AA, French SW (2003). Animal models of alcoholic liver disease; focus on the intragastric feeding model. Alcohol Res Health.

[B23] Stark AH, Madar Z (1993). *In vitro *production of short-chain fatty acids by bacterial fermentation of dietary fiber compared with effects of those fibers on hepatic sterol synthesis in rats. J Nutr.

[B24] Roumes H, Beauvieux MC, Rigalleau V, Gin H, Gallis JL (2006). The decrease of rat liver oxidative phosphorylation induced by butyrate ingestion is under insulin control [abstract]. Magn Res Mat Phys Biol Med.

[B25] Morand C, Rémésy C, Demigné C (1993). Fatty acids are potent modulators of lactate utilization in isolated hepatocytes from fed rats. Am J Physiol.

[B26] Gonzalez-Manchon C, Martin-Requiro A, Ayuso MS, Parrilla R (1992). Role of endogenous fatty acids in the control of hepatic gluconeogenesis. Arch Biochem Biophys.

[B27] Boden G, Chen X (1995). Effects of fat on glucose uptake and utilization in patients with non-insulin-dependent diabetes. J Clin Invest.

[B28] Morand C, Besson C, Demigné C, Rémésy C (1994). Importance of the modulation of glycolysis in the control of lactate metabolism by fatty acids in isolated hepatocytes from fed rats. Arch Biochem Biophys.

[B29] Fukuta Y, Arii K, Matsuda A, Kokuba Y (1998). Comparison of glucose and fat as energy sources in peripheral parenteral nutrition in rats. J Nut Sci Vitaminol.

[B30] Ercan N, Gannon MC, Nuttall FQ (1996). Allosteric regulation of liver phosphorylase a: revisited under approximated physiological conditions. Arch Biochem Biophys.

[B31] Allick G, Bisschop PH, Ackermans MT, Endert E, Meijer AJ, Kuipers F, Sauerwein HP, Romijn JA (2004). A low-carbohydrate/high-fat diet improves glucoregulation in type 2 diabetes mellitus by reducing postabsorptive glycogenolysis. J Clin Endocrinol Metab.

[B32] Paquot N, Schneiter P, Scheen AJ, Lefèbvre PJ, Tappy L (2000). Assessment of postprandial hepatic glycogen synthesis from uridine diphosphoglucose kinetics in obese and lean non-diabetic subjects. Int J Obes Relat Metab Disord.

[B33] Bischof MG, Bernroider E, Krssak M, Krebs M, Stingl H, Nowotny P, Yu C, Shulman GI, Waldhäusl W, Roden M (2002). Hepatic glycogen metabolism in type 1 diabetes after long-term near normoglycemia. Diabetes.

[B34] Aiston S, Andersen B, Agius L (2003). Glucose 6-phosphate regulates hepatic glycogenolysis through inactivation of phosphorylase. Diabetes.

[B35] Rémésy C, Demigné C, Chartier F (1980). Origin and utilization of volatile fatty acids in the rat. Reprod Nutr Dev.

[B36] Schenk S, Davidson CJ, Zderic TW, Byerley LO, Coyle EF (2003). Different glycemic indexes of breakfast cereals are not due to glucose entry into blood but to glucose. Am J Clin Nutr.

[B37] Lau C, Faerch K, Glumer C, Tetens I, Pedersen O, Carstensen B, Jorgensen T, Borch-Johnsen K, Inter99 study (2005). Dietary glycemic index, glycemic load, fiber, simple sugars, and insulin resistance: the Inter99 study. Diab Care.

[B38] Ylonen K, Saloranta C, Kronberg-Kippila C, Groop L, Aro A, Virtanen SM, Botnia Dietary Study (2003). Associations of dietary fiber with glucose metabolism in nondiabetic relatives of subjects with type 2 diabetes: the Botnia Dietary Study. Diabetes Care.

[B39] Feldeisen SE, Tucker KL (2007). Nutritional strategies in the prevention and treatment of metabolic syndrome. Appl Physiol Nutr Metab.

[B40] Kaline K, Bornstein SR, Bergmann A, Hauner H, Schwartz PE (2007). The importance and effect of dietary fiber in diabetes prevention with particular consideration of whole grain products. Horm Metab Res.

[B41] Giacco R, Parillo M, Rivellese AA, Lasorella G, Giacco A, D'Episcopo L, Riccardi G (2000). Long-term dietary treatment with increased amounts of fiber-rich low-glycemic index natural foods improves blood glucose control and reduces the number of hypoglycemic events in type 1 diabetic patients. Diabetes Care.

[B42] Dehghan-Kooshkghazi M, Mathers JC (2004). Starch digestion, large-bowel fermentation and intestinal mucosal cell proliferation in rats treated with the alpha-glucosidase inhibitor acarbose. Br J Nutr.

[B43] Weaver GA, Tangel CT, Krause JA, Parfitt MM, Jenkins PL, Rader JM, Lewis BA, Miller TL, Wolin MJ (1997). Acarbose enhances human colonic butyrate production. J Nutr.

[B44] Song YJ, Sawamura M, Ikeda K, Igawa S, Yamori Y (2000). Soluble dietary fibre improves insulin sensitivity by increasing muscle GLUT-4 content content in stroke-prone spontaneously hypertensive rats. Clin Exp Pharmacol Physiol.

[B45] Galisteo M, Duarte J, Zarzuelo A (2008). Effects of dietary fibers on disturbances clustered in the metabolic syndrome. J Nutr Biochem.

[B46] Guignot L, Mithieux G (1999). Mechanisms by which insulin, associated or not with glucose, may inhibit hepatic glucose production in the rat. Am J Physiol.

[B47] Anand P, Gruppuso PA (2005). The regulation of hepatic protein synthesis during fasting in the rat. J Biol Chem.

[B48] Gautier-Stein A, Zitoun C, Lalli E, Mithieux G, Rajas F (2006). Transcriptional regulation of the glucose-6-phosphatase gene by cAMP/vasoactive intestinal peptide in the intestine. Role of HNF4alpha, CREM, HNF1alpha, and C/EBPalpha. J Biol Chem.

[B49] Cardin S, Emshwiller M, Jackson PA, Snead WL, Hastings J, Edgerton DS, Cherrington AD (1999). Portal glucose infusion increases hepatic glycogen deposition in conscious unrestrained rats. J Appl Physiol.

[B50] Gustafson LA, Neeft M, Reijngoud DJ, Kuipers F, Sauerwein HP, Romijn JA, Herling AW, Burger HJ, Meijer AJ (2001). Fatty acid and aminoacid modulation of glucose cycling in isolated rat hepatocytes. Biochem J.

[B51] Caro JF (1991). Clinical review 26: Insulin resistance in obese and nonobese man. J Clin Endocrinol Metab.

